# Evolution of mantis shrimps (Stomatopoda, Malacostraca) in the light of new Mesozoic fossils

**DOI:** 10.1186/1471-2148-10-290

**Published:** 2010-09-21

**Authors:** Joachim T Haug, Carolin Haug, Andreas Maas, Verena Kutschera, Dieter Waloszek

**Affiliations:** 1 Biosystematic Documentation, University of Ulm, Helmholtzstr. 20, D-89081 Ulm, Germany

## Abstract

**Background:**

We describe new specimens of Mesozoic mantis shrimps (Stomatopoda, Malacostraca) that exhibit morphological and developmental information previously unknown.

**Results:**

Specimens assigned to the taxon *Sculda *exhibit preserved pleopods, thoracopods including all four raptorial limbs as well as details of antennae and antennulae. The pleopods and the antennulae resemble those of the modern mantis shrimps, but the raptorial limbs are not as differentiated as in the modern species. In some specimens, the first raptorial limb (second thoracopod) is not significantly larger than the similar-sized posterior three pairs (as in extant species), but instead these appendages become progressively smaller along the series. In this respect they resemble certain Palaeozoic stomatopods. Another specimen, most likely belonging to another species, has one pair of large anterior raptorial thoracopods, a median-sized pair and two more pairs of small-sized raptorial appendages and, thus, shows a new, previously unknown type of morphology. A single specimen of *Pseudosculda laevis *also exhibits the size of the raptorial limbs; they are differentiated as in modern species, one large pair and three small pairs. Furthermore, we report additional larval specimens and show also post-larval changes, e.g., of the tail fan.

**Conclusions:**

These new data are used to reconsider the phylogeny of Stomatopoda. We still need a strict taxonomical revision of the Mesozoic mantis shrimps, but this first examination already demonstrates the importance of these fossils for understanding mantis shrimp evolution and the interpretation of evolutionary pathways of particular features.

## Background

The Stomatopoda or mantis shrimps are impressive malacostracan crustaceans. They are easily differentiated from other "shrimps" by a number of eye-catching characters. One of these is the structure of their eyes that are differentiated into two halves each, which allows binocular vision with a single eye. The eyes can have up to 12 different colour receptors and are able to detect polarised light [1,2]. Another character is the presence of specific raptorial limbs that can be moved in astonishingly fast and furious strikes [3]. Additionally, stomatopods possess unique designs of their antennulae, antennae and tail fan.

**Figure 1:**
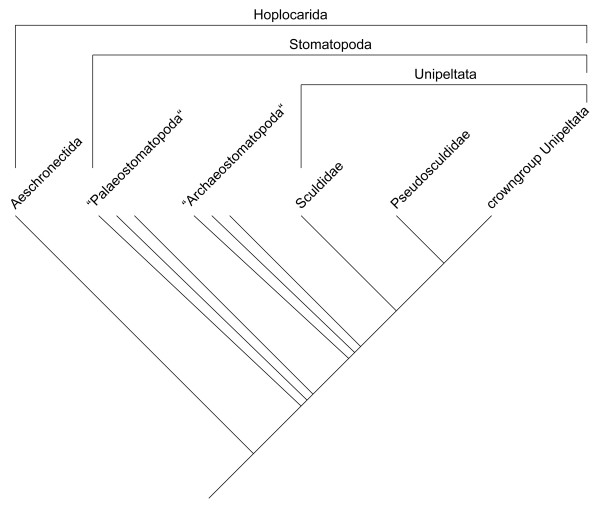
**Proposed phylogeny of Hoplocarida based on all data known before this study**. "Palaeo-" and "Archaeostomatopoda" are written in apostrophes and represented by three parallel branches each, as they are most likely non-monophyletic.

The large morphological gap of the extant Stomatopoda to the other Eumalacostraca (Caridoida) is bridged by a number of fossil groups all belonging to the more comprehensive taxon Hoplocarida, of which the extant stomatopods are the crown group (Figure 1). The Hoplocarida consist of the Aeschronectida and the raptorial hoplocaridans (Stomatopoda) [4]. Aeschronectida comprise species exclusively known from the Carboniferous [5-7]. They possess a tri-flagellate antennula and a relatively enlarged pleon, as do Stomatopoda, but they lack other characteristics of this taxon, most obviously the raptorial appendages.

Stomatopoda comprise the taxa "Palaeostomatopoda", "Archaeostomatopoda" and Unipeltata (Figure 1). Palaeostomatopods have originally been proposed to be the sister group to the rest of the Stomatopoda [6]. The group is now recognised as a paraphyletic assemblage [7,8]. The palaeostomatopods are known from the Carboniferous. They possess sub-chelate raptorial appendages, four sub-equal pairs of more or less the same size.

The archaeostomatopods have been proposed to be the sister group of the Unipeltata (Sculdidae [Mesozoic] + Pseudosculdidae [also Mesozoic] + extant stomatopods). Like the palaeostomatopods they are probably also a paraphylum [8]. Within archaeostomatopods, certain species, e.g., those of the taxon *Tyrannophontes *Schram, 1969, exhibit a differentiation of the raptorial appendages: the posterior three pairs are significantly smaller than the anterior pair, thus they appear comparable to the morphology present in extant stomatopods [8].

The Sculdidae and Pseudosculdidae, along with representatives of a few modern forms, occurred in the Jurassic and Cretaceous [9]. They are united with the crown-group stomatopods in the taxon Unipeltata (Figure 1). Together these species yield significant information in understanding stomatopod evolution, but many details, especially those of the species of *Sculda *Münster, 1840 remain a problem due to insufficient knowledge. While the first raptorial limb of Pseudosculdidae is relatively well known [8,10,11] and resembles that of extant stomatopods [8], details of the raptorial appendages of Sculdidae have not been described yet [[8,10] but see [9,12].

Herein, we present additional data on Sculdidae from the Jurassic Solnhofen Lithographic Limestones, especially details of the raptorial appendages. We also present new details concerning the raptorial appendages of *Pseudosculda laevis *(Schlüter, 1874) from the Cretaceous fish beds of Lebanon. We used these new data to amend earlier computer-based phylogenetic analyses of Stomatopoda [8], to demonstrate how these morphologies "bridge" the gap between Palaeozoic and modern forms, and to present a more precise evolutionary scenario of Stomatopoda.

## Results

### Taxonomic remarks

**Figure 2:**
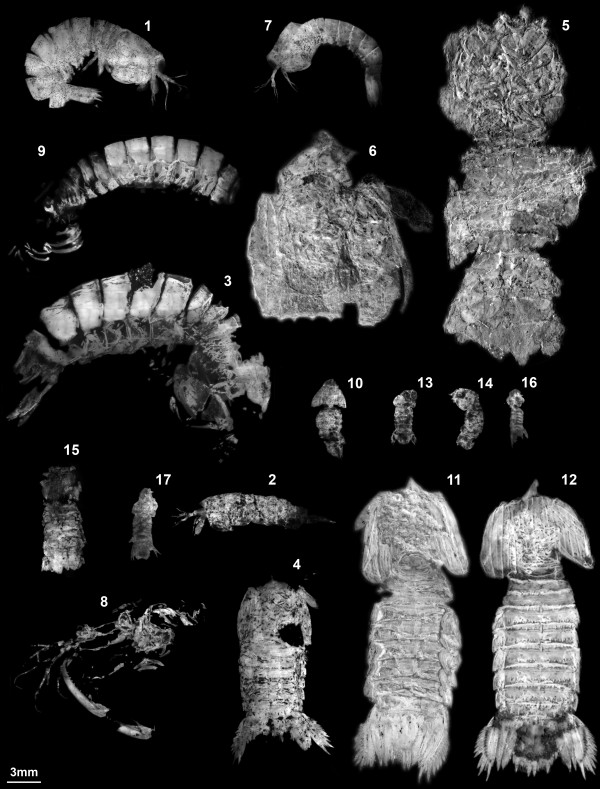
**All specimens used in this study**. All specimens to the same scale to exhibit the size range. Specimens are either shown as inverted images under normal light (no. 5, 6, 11, 12), UV-fluorescence images (no. 2, 4, 13, 14), or green-orange fluorescence images (no. 1, 3 [see also Ref. [17], Figure 3], 7-10, 15-17). Numbers refer to table 1 and to the order, in which the specimens appear on the figures.

The distinction between the three described species of *Sculda *from the Solnhofen Lithographic Limestones (cf. Tab. 1) is not clear and the validity of all these species is at best uncertain, based on our examination of a large number of specimens (part of them depicted in Figure 2). A large-scale taxonomic revision of the group is urgently necessary, but still under way. Therefore, all species names of the taxon *Sculda *- when referring to our material - are given with question marks. *Sculda pennata *Münster, 1840 and *S. spinosa *Kunth, 1870 have been described as differing in regard to rostrum shape and the number of dorsal teeth on the tergites [12]. Both structures are usually not preserved or at least hard to identify in ventral and lateral aspects. Differences in gross shape also have been used to distinguish between the two species, *S. spinosa *being said to be broader than *S. pennata*. Closer investigation of specimens in ventral aspect revealed that these differences might best be interpreted as the result of different degrees of telescoping of the body segments. At the moment we cannot distinguish between the two species, and we believe they might indeed be synonymous. To keep an open terminology, we refer to the specimens that might belong to one of the two species as ?*Sculda pennata/spinosa*.

*Sculda pusilla *Kunth, 1870 is, until now, exclusively known from a single specimen [12-14] (Fig. 2.15). We have two more possible conspecific specimens. *Sculda pusilla *differs from the other two species in having no dorsal surface ornamentation, but is rather smooth [12]. This character causes problems, as all smaller specimens lack such ornamentation, and if this was indeed a reliable diagnostic character for *S. pusilla*, this species might, in fact, be the most abundant one in the Solnhofen Lithographic Limestones. Whether lack of dorsal surface tuberculation is a preservational or an ontogenetic effect remains unclear to us at the moment (possibility of an ontogenetic effect was rejected by Kunth 1870). We refer to the larger specimens (>15 mm) that lack surface ornamentation as ?*Sculda pusilla*, and to small specimens (<15 mm) as *Sculda *sp. (see table 1 for assignment of specimens to morphotypes or species).

**Table 1:** List of all specimens used for this study

**No**.	Species	Light	Location	Collection	Repository number
01	*Sculda sp.*	green	Solnhofen LL, Schernfeld, Lower Tithonian, Hybonotum zone	NW	286

02	*Sculda sp.*	UV	Solnhofen LL, Blumenberg near Eichstätt, Lower Tithonian, Hybonotum zone	MF	---

03	?*Sculda pusilla*	green	Solnhofen LL, Zandt, Lower Tithonian, Hybonotum zone	SMNS, former coll. Ludwig 1992	SMNS 67505

04	?*Sculda pennata/spinosa*	UV	Solnhofen LL, Eichstätt, Lower Tithonian, Hybonotum zone	RF	---

05	?*Sculda pennata/spinosa*	normal	Solnhofen LL, Wattendorf, Lower Kimmeridian, Pseudomutabilis zone	MW	---

06	?*Sculda pennata/spinosa*	normal	Solnhofen Lithographic Limestones (no exact location documented)	SSPHG München	AS I 813

07	*Sculda sp.*	green	Solnhofen LL, Blumenberg near Eichstätt, Lower Tithonian, Hybonotum zone	MW	9308

08	*Pseudosculda laevis*	green	Libanon, Hadjoula, Cenomanian	SSPHG München	1967 I 334

09	?*Sculda pusilla*	green	Solnhofen LL, Zandt, Lower Tithonian, Hybonotum zone	RF	---

10	*Sculda sp.*	green	Solnhofen LL, Wegscheid near Schernfeld, Lower Tithonian, Hybonotum zone	NW	---

11	?*Sculda pennata/spinosa*	normal	Solnhofen LL, Breitenhill/Öchselberg, Upper Kimmeridgian, Beckeri zone	MW	9701

12	?*Sculda pennata/spinosa*	normal	Solnhofen LL, Zandt, Lower Tithonian, Hybonotum zone	RF	---

13	*Sculda sp.*	UV	Solnhofen LL, Blumenberg near Eichstätt, Lower Tithonian, Hybonotum zone	MF	---

14	*Sculda sp.*	UV	Solnhofen LL, Breitenhill/Öchselberg, Upper Kimmeridgian, Beckeri zone	MW	1995

15	*Sculda pusilla*	green	Solnhofen Lithographic Limestones (no exact location documented)	SSPHG München	AS I 814

16	*Sculda sp.*	green, normal	Solnhofen LL, Zandt, Lower Tithonian, Hybonotum zone	PR	87133

17	*Spinosculda ehrlichi*	green	Solnhofen LL, Zandt, Lower Tithonian, Hybonotum zone	MW	9203

Each specimen in the table is depicted in Figure 2 in the same numerical order. Collections: MF = Michael Fecke, Langenberg; MW = Matthias Wulf, Rödelsee; NW = Norbert Winkler, Stahnsdorf; PR = Peter Rüdel, Gröbenzell; RF = Roger Frattigiani, Laichingen; SMNS = Staatliches Museum für Naturkunde Stuttgart; SSPHG München = Staatliche Sammlung für Paläontologie und Historische Geologie München.

### Description of structures

#### Antennula (Figure 3A, B)

**Figure 3:**
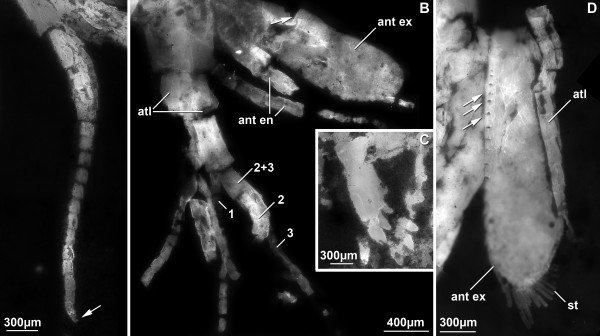
**Details of anterior appendages of *Sculda *Münster, 1840**. A. Complete antennular flagellum of a specimen of *Sculda *sp. (no. 1). The rounded shape of the most distal annulus (arrow) indicates that it is indeed the most terminal one. B. Antennula and antenna of a specimen of *Sculda *sp. (no. 2). Note the branching pattern of the antennula and the division of the antennal exopod paddle (arrow). Numbers refer to the three flagella of the antennula. C. Mandibles of a specimen of ?*Sculda pusilla *(no. 3). D. Antenna of a specimen of ?*Sculda pennata/spinosa *(no. 4). Arrows point to insertions of setae. Abbreviations: ant en = antennal endopod, ant ex = antennal exopod, atl = antennula, st = setae.

The antennulae are tri-flagellate. The basal part, the peduncle comprises three tubular articles. These articles are about twice as long as wide. The proximal flagellum branches off from the second article, the distal two flagella arise form the third article. The highest number of annuli in a flagellum is 15. Annuli are about as long as wide.

#### Antenna (Figure 3B, D)

Proximal parts are unknown, they are usually concealed by the shield ("carapace"). The endopod is flagellate, made up of more than ten annuli, the maximum number is probably higher. The exopod is a paddle and appears to have two parts: the proximal part of the exopod is more or less square in antero-posterior aspect, the distal part of the exopod is paddle-shaped and about three times as long as wide (Figure 3B). In one specimen of ?*Sculda pennata/spinosa *the entire margin of the distal part is fringed with at least 32 small setae. Where the setae are missing, they are still indicated by their small sockets (Figure 3D). Of these 32 setae, about 20 setae are situated along the medial margin, the remaining twelve are around the distal aspect. Whether the lateral margin is also fringed by small setae remains unclear. The proximal diameter of the setae is about 1/20 of the maximum width of the exopod paddle. The maximum preserved length of the setae is about twelve times the width, but based on their shape, they were probably significantly longer.

#### Mandible (Figure 3C)

The mandible is preserved in a single specimen of ?*Sculda pusilla*. Only the coxal body is preserved, no traces of a palpus are apparent; whether this is due to preservation or represents the true morphology is not known (both conditions are present in extant species). The coxal body is at least three times as long (latero-median axis) as high (proximo-distal axis) and is drawn out medially into four stout finger-like spines.

The maxillula, maxilla and first maxilliped (grooming limb) are not preserved in a single specimen. Therefore, their morphology remains unknown.

#### Raptorial limbs (Figure 4, 5A, B)

**Figure 4:**
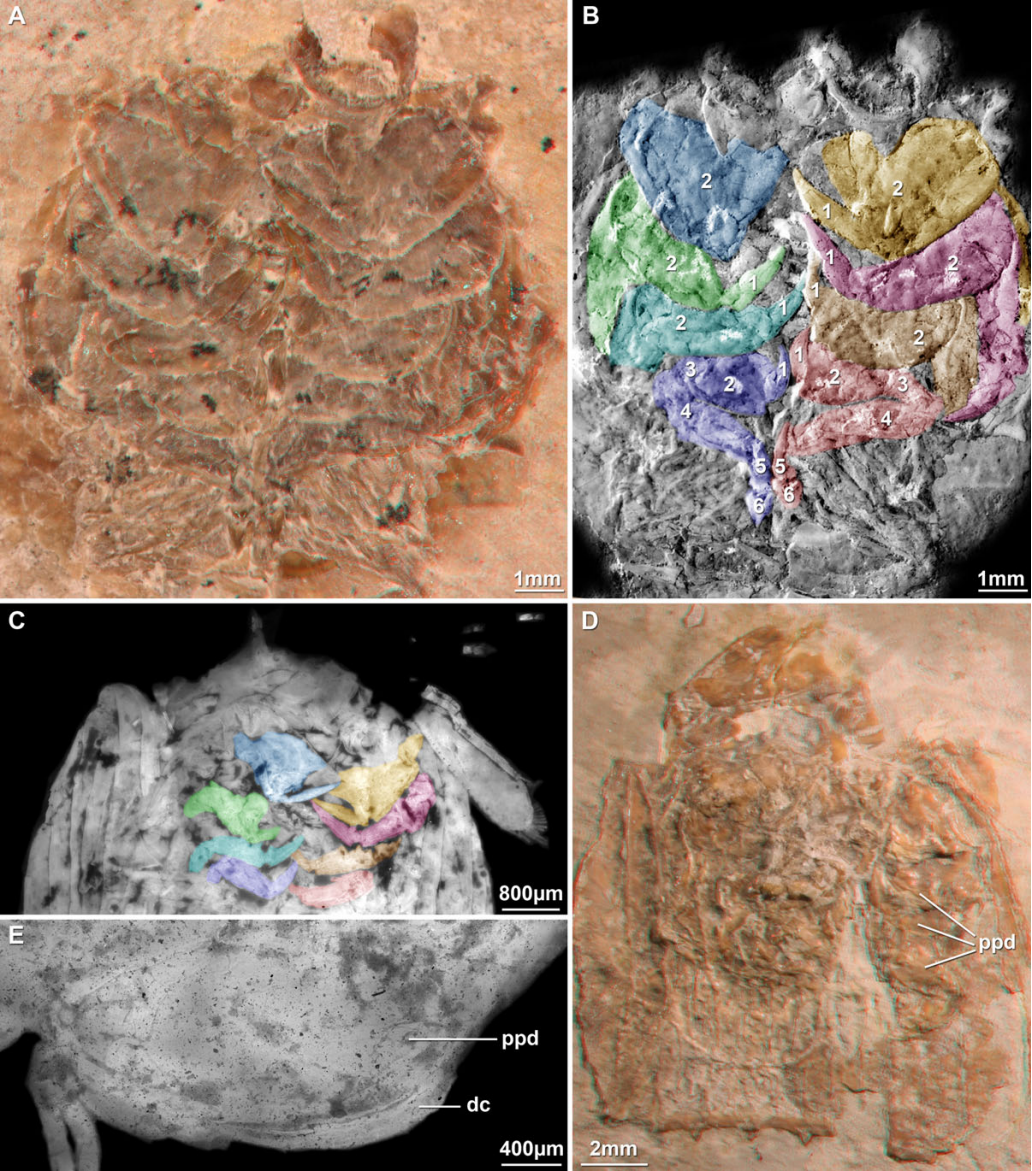
**Details of raptorial appendages**. A. Stereo image of the anterior part of a specimen of ?*Sculda pennata/spinosa *(no. 5) exhibiting details of the raptorial thoracopods. B. Colour-coded image of the same area as in A. Image recorded under normal light, then inverted. Each raptorial limb is marked in a different colour. Numbers mark the different articles of the limb. C. UV-composite-fluorescence image of the anterior region of a smaller specimen of ?*Sculda pennata/spinosa *(no. 4). Colours as in B. D. Stereo image of an isolated anterior part of a specimen of ?*Sculda pennata/spinosa *(no. 6). This specimen is one of the original specimens of Kunth (1870) described as belonging to *Sculda spinosa *Kunth, 1870. Note the preserved raptorial thoracopods. E. Second thoracopod of a specimen of *Sculda *sp. (no. 7) preserved in lateral aspect. Abbreviations: dc = dactylus, ppd = propodus.

**Figure 5:**
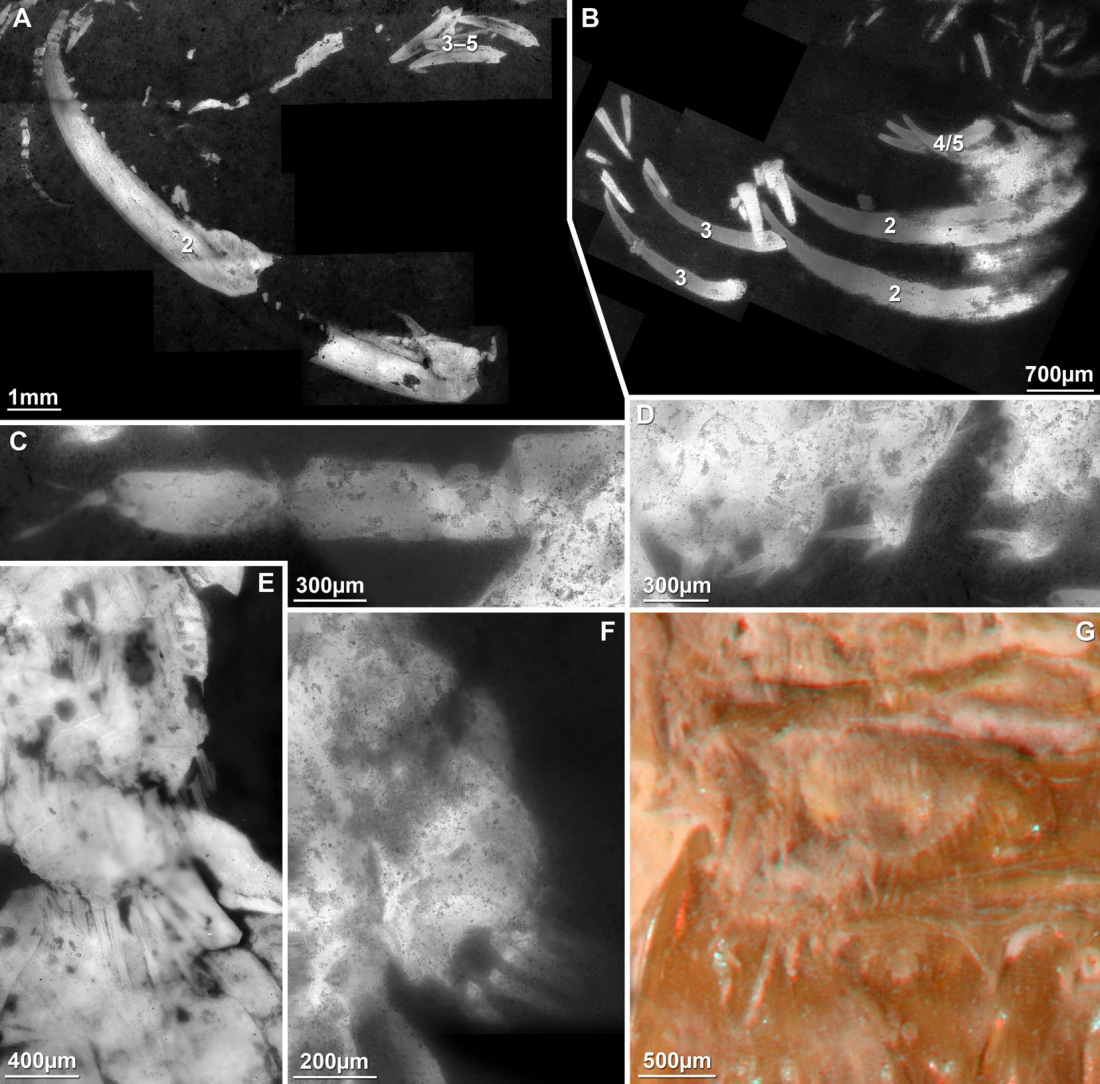
**Details of thoracopods (continued from Figure 4) and pleopods**. A. Anterior part of a specimen of *Pseudosculda laevis *(Schlüter, 1874) (no. 8) in lateral view. Note the teeth on the large dactylus (2) and the size difference to the posterior three dactyli (3-5) of the thoracopods. B. Dactyli of the thoracopods of a specimen of ?*Sculda pusilla *(no. 9). Note the size difference between the large-sized second thoracopod (2), the medium-sized third (3), and the small-sized fourth and fifth ones (4/5). C. Posterior thoracopod, i.e., walking limb of a specimen of *Sculda *sp. (no. 1). D. Pleopods of a specimen of *Sculda *sp. (no. 1) in lateral view. No details apparent. E. UV-composite-fluorescence image of a specimen of ?*Sculda pennata/spinosa *(no. 4), details of the pleopods in ventral view. F. UV-composite-fluorescence image of a specimen of *Sculda *sp. (no. 10), detail of the single preserved pleopodal exopod in dorsal view. Note the smaller number of setae compared to E. G. Red-cyan stereo image of a specimen of ?*Sculda pennata/spinosa *(no. 11), detail of a right pleopod in ventral view. Note how the paddle-shaped endopod is smoothly bent.

In general, describing raptorial limbs is difficult because of terminological issues. The raptorial thoracopods, i.e., the thoracopods 2-5, have only six articles, while the thoracopods of other eumalacostracans have seven articles (= podomeres). As Schram [8] has pointed out, without an exopod as reference point to identify the basipod the exact identity of the six articles remains unclear. There is a consensus that the most distal article is termed dactylus, the next one propodus and the third one carpus. From here on the terminology is no longer uniform [8]. We restrict ourselves in the following to naming only the distal three parts and numbering the remaining parts consecutively, including the three distal ones. As in most specimens only the distal parts of the limbs are preserved, counting starts with the dactylus = 1, propodus = 2, carpus = 3, etc.

This terminology is far from satisfying, but as neutral as possible, without losing clarity. The homology of the so-called dactylus to the dactyli of other Malacostraca may be also questioned. Nevertheless, this term is unavoidable here. How to describe a sub-chelate claw of a stomatopod without referring to dactylus and propodus? Yet, one has to keep in mind that these terms are used here in a functional sense and do not necessarily imply homologies to other malacostracans. The exact identity of the articles of the raptorial limbs is an important issue to be solved in the future.

For further distinguishing of the different morphotypes, the size patterns of the four raptorial appendages along the series are given as ratios of dactylus lengths (for comparison with other species see Additional file 1).

The dactylus of the second thoracopod is the longest. The formula for dactyli lengths within one raptorial apparatus is:

2nd thp : (3rd thp/2nd thp) : (4th thp/2nd thp) : (5th thp/2nd thp). [thp = thoracopod]

The length of the dactylus of the second thoracopod is set as 1, consequently all following lengths are 0.xx.

Three different types of raptorial patterns are recognised within the investigated material. The first pattern is exclusively seen on specimens of ?*Sculda pennata/spinosa *(Figure 4A-D). The four pairs of appendages are, more or less, sub-equal, their sizes decreasing progressively along the series. The lengths of the dactyli are 1 : 0.87 : 0.47 : 0.40. The dactyli are smooth, i.e., lacking any teeth and serrations and form sub-chelae against the disto-median edge of the propodus. The propodi are sub-oval in antero-posterior outline, very massive and large, at least twice as long as the dactyli and wider than the dactyli are long. The carpus is best known for the fifth thoracopod; it is short and almost triangular. On the more anterior limbs, the carpus appears to be progressively longer on each further anterior limb, but it is in fact difficult to judge because of the preservation. The more proximal parts are exclusively known for the fifth thoracopod. Article 4 is relatively long, about four times as long as wide. Article 5 is slimmer than 4, about half the width, and also about four times as long as wide. Article 6 is rather small and as long as wide. Massive propodi with blade-like dactyli can also be observed in *Sculda *sp, i.e., in small specimens (Figure 4E). The more posterior raptorial appendages are unfortunately not visible in these specimens.

The second pattern is recognised as different from the first type based on the lengths of the dactyli. It is exhibited by one specimen of ?*Sculda pusilla *(Figure 5B). The dactyli are smooth; they appear blade-like and are gently curved. The largest pair of dactyli (here interpreted as that of the second thoracopod) is more or less twice as large as the second largest pair (here interpreted as that of the third thoracopod), while the other two dactyli (that of the fourth and fifth thoracopods) are significantly smaller. Therefore, the formula is 1 : 0.5 : 0.29 : 0.24. The shapes of the propodi are mostly unknown. For the second thoracopods the propodi appear to be more or less oval-shaped (Figure 2.3). The more proximal parts are not preserved.

The third type is exhibited by an ill-preserved specimen of *Pseudosculda laevis *(Figure 5A). Like the second type it is also exclusively recognised based on the dactyli lengths. The dactyli are, as for ?*Sculda pusilla*, long, curved and blade-like. Unlike ?*S. pusilla*, at least the largest dactylus appears to have eight small teeth along the inner side. The first pair of dactyli is more than three times as long as the sub-equal three pairs of posterior ones, expressed by 1 : 0.29 : 0.27 : 0.27. All proximal parts are unknown.

#### Walking limbs (Figure 5C)

The morphology of the walking limbs (sixth to eighth thoracopod) can only be observed on a single specimen of *Sculda *sp. The whole limb appears to be tubular, with at least four articles. The distal part is twice as long as wide and tapers distally. It carries two distally directed setae, which are about one fourth of the article wide and only slightly shorter than the article in length. The next proximal article is of the same shape as the distal one, but slightly larger. It tapers distally. A small seta arises disto-medially, which seems to be about half the length of the setae of the distal article. The next proximal article is stouter, the width about the same as the penultimate article, but it is significantly shorter, having only about one third of the length of the penultimate article. The next proximal article is only poorly known, as it is concealed by the body. The article appears to be massive, about 1.5 times the width of the next distal article, and at least as long as wide.

#### Pleopods (Figure 5D-G)

The detailed morphology of the pleopods can be observed in specimens of ?*Sculda pennata/spinosa *(Figure 5E, G) and specimens of *Sculda *sp. (Figure 5D, F). The proximal part, the basipod, is difficult to recognise. Based on the ventral foramen it is at least twice as wide in latero-median axis than in anterior-posterior axis. The proximo-distal axis of the basipod can only be indirectly judged, but appears to be no more than half of the width. The exopod is paddle-shaped with at least ten setae along the distal margin and about twelve tooth-like outgrowths along the lateral margin. The endopod is also paddle-shaped, its distal margin carries at least twelve setae. Exopod and endopod are about the same size. Smaller specimens (*Sculda *sp.) only exhibit incomplete pleopods, thus the exact morphology of the earlier developmental stages remains unclear. There are fewer setae on the exopod, about seven in a 0.7 mm large specimen, possibly also on the endopod compared to later stages.

#### Uropods (Figure 6A, D, G, H)

**Figure 6:**
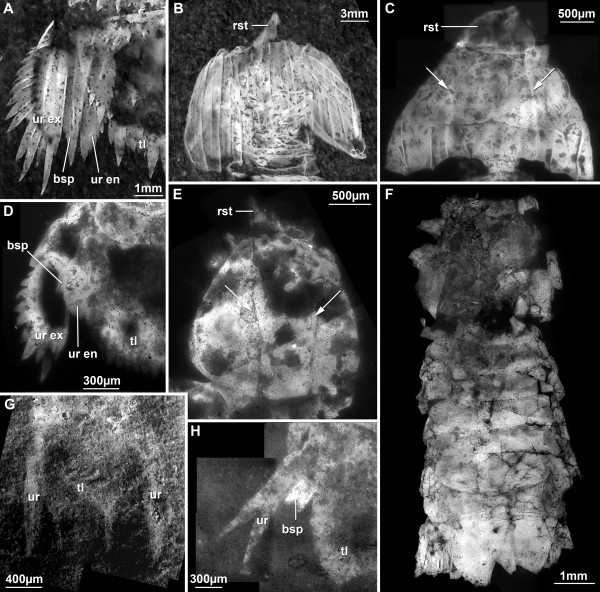
**Details of uropods, telson and shield**. A. Left uropod and left part of telson of a specimen of ?*Sculda pennata/spinosa *(no. 12) in dorsal view. B. Head shield of a specimen of ?*Sculda pennata/spinosa *(no. 12). Note the ridges running antero-posteriorly. C. Head shield of a specimen of *Sculda *sp. (no. 10). The division of the shield into three fields is apparent (see arrows), but the ridges are only weakly expressed. D. Left uropod and left half of the telson of a specimen of *Sculda *sp. (no. 13). E. Head shield of a specimen of *Sculda *sp. (no. 14), showing the tripartite-division (arrows). F. Holotype of *Sculda pusilla *Kunth, 1870 (no. 15). Not many details preserved. G. Uropods and telson of a specimen of *Sculda *sp. (no. 16). The image used here was taken under normal light, whereas the corresponding image in Figure 2 was taken under green light. H. Part of telson and one uropod of one specimen of *Spinosculda ehrlichi *Haug, Haug & Waloszek, 2009 (no. 17). Abbreviations: bsp = basipodal spine, rst = rostrum, tl = telson, ur = uropod, ur en = uropodal endopod, ur ex = uropodal exopod.

Different morphotypes of uropods can be found on several specimens of *Sculda*. We interpret these as developmental differences, because two are exclusively observed on very small specimens; another one is present exclusively on larger specimens. The uropods of the largest morphotype present in specimens of ?*Sculda pennata/spinosa *are composed of the basipod ("protopod"), the exopod and the endopod (Figure 6A). The most proximal aspect of the basipod is not exactly known. The basipodal process ("protopodal" process) also characteristic for extant stomatopods is long, blade-like, and forked, but not symmetrical, i.e., there is a large distal and a small lateral tip. The endopod is long and paddle-shaped with ten teeth or strong serrations along the median margin, one lateral serration and a tooth-like distal tip. The exopod is also long and paddle-like. Along the lateral margin there are 14 movable spines present; additionally, 14 immovable tooth-like serrations are present dorsal to the movable spines. Two of these, the ones left and right of the distalmost movable spine, are enlarged and appear spine-like. A ridge runs from proximal to distal on the surface of the exopod, dividing at about one third of the width of the lateral part of the exopod.

A smaller morphotype of uropod, which we interpret as an earlier developmental stage in specimens of *Sculda *sp. of about 0.5 mm in length, differs in certain aspects (Figure 6D). The basipodal spine is very small and is not bifurcate. The endopod is also paddle-shaped, but instead of having well-developed teeth, there are only three serrations on its distal end. The exopod has only nine movable teeth and eight immovable dorsal serrations (including the two spine-like distal ones). Both teeth and serrations appear stouter than those found in the larger developmental stage.

A single very small and incompletely preserved specimen may be a larva of *Sculda *sp. (Figure 6G). Its uropods appear to have elongate lanceolate endopods and exopods without any teeth or setae, but more than a vague outline is not identifiable.

A single new specimen of *Spinosculda ehrlichi *Haug, Haug & Waloszek, 2009 is very small and shows no really new details (Figure 6H), but is significantly smaller than the holotype, a late larval stage. The new specimen is interpreted as an earlier larval stage, the third known developmental stage and earliest of this species. Exopod and endopod are both spine-like without setae or spines; both rami are sub-similar. The basipodal spine is better preserved than in the holotype [15]. It is apparently almost symmetrically forked.

#### Telson (for a discussion of the difficulties with the term 'telson' within Malacostraca see [16]) (Figure 6A, D, G, H)

The morphology of the telson of larger specimens is very similar to that described in the literature [12]. The main new details are 19 dorsally drawn out serrations above the 18 movable teeth, comparable to those on the exopods of the uropods (Figure 6A).

Smaller specimens exhibit slight differences that are interpreted as developmentally caused (Figure 6D). A differentiation of tooth length is already indicated. A pair of central spines, as well as the sixth ones (counted from the middle), is slightly larger than the other spines. In later stages these are at least twice as long as the others. The possible larval specimen possesses a bifurcate telson without any indications of teeth (Figure 6G).

The telson of the new specimen of *Spinosculda ehrlichi *is bifurcate, as for the older known stages, but not as differentiated as in the later stages (Figure 6H). It also lacks the initial additional spines present on the holotype, which represents a later larval stage [15].

#### Shield ("carapace") (Figure 6B, C, E)

The shield of larger specimens in later developmental stages does not differ from that described in the literature [12] (Figure 6B). Smaller specimens, i.e., earlier developmental stages, exhibit certain differences that are interpreted as developmentally caused. The smallest specimens have a shield that is obviously tri-fold with a central and two lateral areas as in the later stages, but lacks the prominent ridges that run from anterior to posterior (Figure 6E). A slightly larger specimen of *Sculda *sp. also has the three areas developed. It additionally shows already some anterior-posterior ridges, but it lacks the grooves running from left to right that are present on the adult shield (Figure 6C). The triangular shape of the rostrum appears to be unchanged during juvenile development.

Unfortunately, neither the shield morphology nor any other morphological details can be clearly determined in the holotype of *Sculda pusilla *(Figure 6F). Probably during the preparation process about 140 years ago all details along the margins of the specimen have been destroyed.

### Phylogenetic analysis

**Figure 7:**
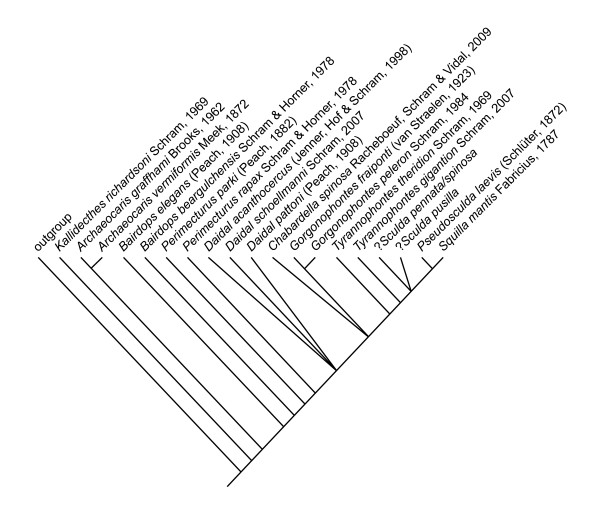
**Results of the phylogenetic analysis run in 'PHYLIP pars'**. Single shortest tree.

The single resulting shortest tree of a total length of 76 steps of the phylogenetic analysis run in 'PHYLIP pars' is given in Figure 7.

## Discussion

### Newly discovered morphological details

A number of morphological details on the species of *Sculda *have not been known before. The antennulae have been supposed to be tri-flagellate because of the hoplocaridan affinities of *Sculda*. We can now present the exact branching pattern and the maximum number of annuli per flagellum. The branching pattern is similar to that known from extant stomatopods and the recently described *Spinosculda ehrlichi *[15] with two distal flagella arising from one article, which itself arises from the same article as the third flagellum.

Kunth [12] depicted the antennae of *Sculda spinosa *in his drawings including setae along the outer margin of the exopod paddle. Whether this was truly visible on his specimens or adapted from knowledge of extant stomatopods remains unclear. We now see the exact setation pattern (partially based on insertions) and the setae themselves. A single specimen also shows the division of the exopod paddle. The annulation of the endopod is also evident, although the distal part remains unknown.

The mandible can, despite its concealed position, also be documented for the first time. In some extant stomatopods the mandible carries the typical tripartite malacostracan palp, in other species it is palpless. Whether absence of a palp in ?*Sculda pusilla *is due to preservation or reflects the true morphology remains unclear.

The maxillula, maxilla and the first thoracopod ("maxilliped") are still unknown. For all three appendages this is due to their concealed position. The more posterior thoracopods and the raptorial appendages, are now known quite well and reveal some unexpected details (see below). The posterior thoracopods, i.e., walking limbs, are also presented for the first time, but appear to be comparable to those of extant stomatopods. The pleopods have already been depicted by Kunth [12], but could now be documented with more details, i.e., the exact number of setae. The uropods of the studied material yielded little new information, except for certain details of the spine pattern. Especially which spines are movable and which not is now better understood.

Early developmental changes could also be documented. In particular, the pattern of spines on the uropods appears to change during post-larval development. Other changes could be documented for the shield and the pleopodal exopods. Such post-larval changes have not been documented for extant species or other fossils, but have the potential to yield information of phylogenetic significance as soon as comparable data are available.

### Phylogenetic significance

**Figure 8:**
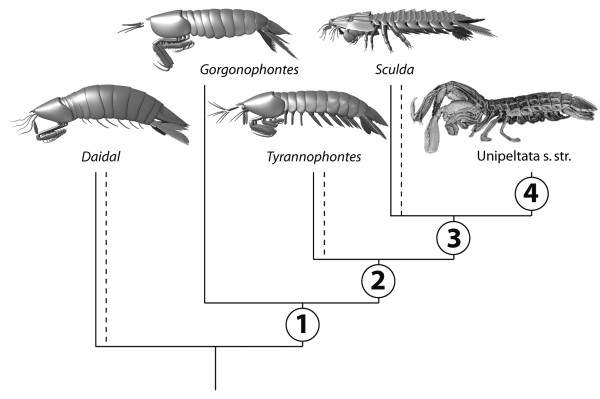
**Proposed phylogeny and evolutionary scenario of an ingroup of Stomatopoda including "Archaeostomatopoda"**. Taxa presented as 3D models based on [[8,20], this paper] besides the representative of Unipeltata sensu stricto, which is a volume rendering of a CT scan. Autapomorphies at the marked nodes: 1: Size reduction of raptorial limbs two, three and four; 2: (Unipeltata sensu lato) Further size reduction of raptorial limbs three and four; 3: Shape change of telson from elongate-triangular to rounded with bisymmetrically arranged teeth/spines; 4: (Unipeltata sensu stricto = Verunipeltata taxon nov. + *Pseudosculda laevis*) Further size reduction of raptorial limb two to the same size as three and four.

The significant insights we gained are not only details of the raptorial appendages of Sculdidae, but also the additional data on the posterior raptorial appendages of *Pseudosculda laevis*. Both have been reported as completely unknown for sculdids and partially known for pseudosculdids [8,10]. Based on these findings, the phylogenetic analysis performed by Schram [8] was amended and rerun, resulting in a better resolution of the Mesozoic species and some changes in the branching pattern leading to a different reconstruction of the evolutionary scenario of stomatopod phylogeny (Figure 8).

Schram [8] has already argued for a close relationship of Pseudosculdidae to the crown group of Unipeltata, although in his analysis the Pseudosculdidae, Sculdidae and crown-group Unipeltata form an unresolved trichotomy, probably a result of the incomplete knowledge of the morphology of the Sculdidae. Our findings, especially concerning the three pairs of posterior raptorial appendages of *Pseudosculda laevis*, support Schram's view that *P. laevis *is the sister species of the crown group of Unipeltata.

The monophyly of Pseudosculdidae has been questioned [10]. Unfortunately, our knowledge of *Archaeosculda phoenicia *Ahyong, Garassino & Gironi, 2007, the second described pseudosculdid species, is not yet detailed enough to include it into our analysis. The new methods for investigating fossils from the Cretaceous fish beds of Lebanon with the aid of orange-green fluorescence [17] (Figure 4A) might also facilitate a re-investigation of the single known specimen of *A. phoenicia *to obtain more details. Also a re-investigation of *P. laevis *appears to hold promise [8,17].

In addition, four other reports of fossil specimens from the Mesozoic are assigned to *Pseudosculda*, all without further investigations and also without concrete taxonomical descriptions. Hof [9] mentioned new specimens from the Jurassic of Osteno, Italy, that are most likely pseudosculdids. Feldmann et al. [18] illustrated specimens from the Cretaceous of Colombia, which they interpreted as *Sculda *sp., but Schram and Müller [14] re-interpreted the material as *Pseudosculda *sp. Ahyong et al. [10] rejected the two interpretations. Further interesting specimens loosely assigned to Pseudosculdidae are from the Cretaceous of Mexico [11]. Moreover, Förster [19] mentioned a specimen representing *Pseudosculda *from the Solnhofen Lithographic Limestones (unfortunately, the specimen has not yet been found in the Staatliche Sammlung für Paläontologie und Historische Geologie München, into which Förster's collection were transferred). All these specimens need to be further investigated before the monophyly of Pseudosculdidae can be verified.

In order to establish a clearer terminology of the taxa involved, we want to propose some new names. The crown group containing all living species often referred to as "extant unipeltatans" [8] should be more exactly termed Verunipeltata taxon nov. Together with the sister group *Pseudosculda laevis *(as long as the status of Pseudosculdidae is unclear) they form a taxon for which we propose the name Unipeltata sensu stricto.

The Sculdidae appear in our phylogenetic analysis as sister group to the Unipeltata sensu stricto, as already supposed by Schram [8], but it remains unclear whether Sculdidae/*Sculda *is a monophylum or a paraphylum. This is caused by the partly incomplete knowledge of the morphotype here referred to as ?*Sculda pusilla*. The Sculdidae are united with Unipeltata sensu stricto by details of, for example, the telson morphology. In *Sculda *specimens the telson is more similar to that of extant stomatopods, i.e., it appears stouter and with teeth arranged in bisymmetrical pairs, whereas the shape in palaeo- and archaeostomatopods is more or less elongate triangular with a median unpaired process. Consequently, a stout telson with teeth arranged in pairs seems to be a (autapomorphic?) ground-pattern character of the unnamed taxon Unipeltata sensu stricto + *Sculda*.

Additionally, the issue of the monophyly of Sculdidae is complicated by the fact that the taxonomic identity of the species from Solnhofen (*Sculda spinosa *and *S. pennata*) needs still to be clarified. Also the still incomplete knowledge of *S. pusilla *(holotype illustrated in Figure 6F) and the species from Lebanon, *S. syriaca *Dames, 1886, further complicate this issue. Interestingly, all specimens we found in private collections labelled as *S. syriaca *turned out to be *Pseudosculda laevis*, the type material of both species is lost according to Schram and Müller [14].

The newly described species *Spinosculda ehrlichi *was also assigned to Sculdidae [15]. As the morphology of its raptorial apparatus is almost unknown, it could not be included into the present analysis. A developmental character based on the newly presented younger larva of the species might give a hint for placing this species closer to Unipeltata sensu stricto than the species of *Sculda*. In ?*Sculda pennata/spinosa *the basipodal spine is still small in early post-larval stages and gains size during later post-larval development. In extant stomatopods the basipodal spine is already present and relatively large in larval stages compared to the exopod and endopod [20]. In *Sp. ehrlichi *a possible basipodal spine was described in the holotype specimen that represents a late larval stage [15]. The newly presented earlier larval stage clearly possesses such a (forked) spine. This character, i.e., the presence of a well developed basipodal spine already in larval stages, may unite *Sp. ehrlichi *and Unipeltata sensu stricto, but this remains an assumption until the Mesozoic stomatopods have been further investigated, e.g., the development of Pseudosculdidae.

Our findings demonstrate that there are not only two major statuses of the raptorial appendage patterns, namely four rather undifferentiated sub-chelate appendages versus one large followed by three sub-equal small appendages. Additionally, there are other possible morphologies as exhibited by the raptorial apparatuses developed in *Sculda*. For comparison and coding of the lengths of the raptorial appendages into the phylogenetic analysis for the Palaeozoic species we had to rely on published images and mainly on the propodi lengths (which appear to be more or less directly correlated to dactyli lengths, but often they are incomplete, thus lengths remain estimations). Interestingly, *Tyrannophontes theridion *Schram, 1969 appears to have a pattern that is similar to that present in ?*Sculda pusilla *in having a second raptorial appendage larger than the third one (cf. [8], his Figure 3). Although this demands a direct re-investigation of the original material, our work shows the presence of one large, one medium-sized and two smaller raptorial limbs (although not as apparent as in our material). We have decided to slightly alter the reconstruction of *T. theridion *(Figure 8) to fit this observation.

For *Tyrannophontes gigantion *Schram, 2007 this issue is less clear. The species is obviously very important for understanding stomatopod evolution based on its resolved phylogenetic position, but the single existing specimen is only imperfectly known [8]. *Tyrannophontes *appears in our analysis as a paraphyletic assemblage, but as Schram [8] has pointed out, this may be due to the lack of knowledge of the pleon of *T. gigantion*. For the taxon comprising Unipeltata sensu stricto, Sculdidae and *Tyrannophontes *we designate Unipeltata sensu lato. This taxon is mainly characterized by the degree of differentiation of the raptorial apparatus and also by the jack-knifing point and the enlarged 4th part of the raptorial appendages. Possibly also the presence of paddle-shaped exopods of the pleopods may characterise this node, while those known from the other archaeostomatopods are multi-annulated [21]. Additionally, the number of annuli in the antennular flagella appears to be lower than in other archaeostomatopods. The branching pattern of the three flagella of the antennulae may also possibly characterise the node of Unipeltata sensu lato. As we could demonstrate for ?*Sculda pennata/spinosa*, the flagella branch as in modern stomatopods, two distal flagella from one article, which arises from another article together with a third flagellum. For other archaeostomatopods, the antennula is often reconstructed with all flagella arising from one article [5,21]. Unfortunately, the morphologies of the critical taxa, namely *Gorgonophontes *Schram, 1984 and *Tyrannophontes *are only incompletely known.

According to our analysis, *Gorgonophontes *or the quite recently described *Chabardella spinosa *Racheboeuf, Schram & Vidal, 2009 from the Carboniferous of France are the sister group to Unipeltata sensu lato. This unresolved polytomy is partly explained by the rather incomplete knowledge of *C. spinosa*, especially of the tail fan area [22]. This result contradicts the analysis of Schram [8], in which *Gorgonophontes *was sister group to *Daidal *Schram, 2007 + Unipeltata sensu lato. *Gorgonophontes *already possesses a slight differentiation of the second thoracopod, which is enlarged compared to the more posterior ones. This differentiation is missing in *Daidal*, which turns out to be the sister group of *Gorgonophontes *+ *C. spinosa *+ Unipeltata sensu lato in our analysis. Thus, the scenario reconstructed here, although differing from Schram's [8], is seen as plausible as Schram [8] has already indicated. The monophyly of the taxon *Daidal *is equivocal, which may be due to the incomplete knowledge of *Daidal pattoni *(Peach, 1908).

The branching pattern of some of the palaeostomatopods differs from Schram's results [8], but *Archaeocaris *Meek, 1872 also results in our analysis as the sister group to all remaining stomatopods. The next basal branchings in Stomatopoda, i.e., the exact relationships of the species of *Bairdops *Schram, 1979 and *Perimecturus *Peach, 1908, are resolved in a different manner. In Schram [8], they form a monophylum, in our result they are a paraphyletic assemblage.

In any case both genera appear as paraphyletic. If the genera *Perimecturus *and *Bairdops *indeed prove to be non-monophyletic, as not only indicated by our analysis, but especially by Schram [8], new names for monophyletic taxa will need to be erected. The same consequent application of phylogenetic systematics also means abandoning the taxa "Archaeostomatopoda" and "Palaeostomatopoda", as both have appeared as paraphyla in various analyses [[7,8], this analysis].

The significant new systematic findings may be summarised like this (basal branchings and taxonomic issues of certain groups excluded):

Unipeltata Latreille, 1825 sensu lato

*Tyrannophontes theridion *Schram, 1969

NN1

*Tyrannophontes gigantion *Schram, 2007

NN2

Sculdidae Dames, 1886

Unipeltata Latreille, 1825 sensu stricto

      Pseudosculdidae Dames, 1886

      Verunipeltata taxon nov.

### Evolutionary scenario

Based on the resulting phylogeny and the known morphologies, the following series of evolutionary changes is proposed (Figure 8). Four sub-similar-sized sub-chelate thoracopods appear to be apomorphic for Stomatopoda and are plesiomorphically retained up to the node of *Daidal *+ (*Gorgonophonthes *+ Unipeltata sensu lato), as for example exhibited by *D. schoellmanni *Schram, 2007 (the incompletely known *Chabardella spinosa *is excluded here). In a first evolutionary change in the direct stem lineage of *Gorgonophontes *+ Unipeltata sensu lato, the first raptorial appendage is slightly enlarged, but the more posterior ones remain sub-equal. This morphology is, for example, exhibited by *G. fraiponti *(van Straelen, 1923). In a next evolutionary change the first raptorial appendage is enlarged while the second (remains?) more or less middle-sized and the third and fourth ones become significantly smaller at the node of Unipeltata sensu lato. This morphology appears to be exhibited by *Tyrannophontes *(especially *T. theridion*, less apparent in *T. gigantion*) and best in ?*Sculda pusilla*. In a last step the second raptorial appendage also becomes significantly smaller than the first pair at the node of Unipeltata sensu stricto, as for example exhibited by the extant *Neogonodactylus bredini *(Manning, 1969) or the well-known *Squilla mantis *Fabricius, 1787.

The raptorial apparatus exhibited by ?*Sculda pennata/spinosa *appears to be autapomorphic, but derived from one like that exhibited by ?*Sculda pusilla*. The exact use of the characteristic massive propodi with the small, but sharp-appearing dactyli must remain in question. In extant stomatopod species such morphology is not developed. The closest resemblances may be the sub-chelate gnathopods of amphipods. They also possess massive propodi, although not as hypertrophied as we have found them in ?*Sculda pennata/spinosa*. Furthermore, they are orientated upside-down, therefore do not facilitate exact comparisons. Similarly orientated raptorial limbs with a large propodus and small dactylus may be present in *Kellibrooksia macrogaster *Schram, 1973 (Malacostraca: Phyllocarida: Hoplostraca) (but see [23]). The large massive propodi of ?*Sculda pennata/spinosa *might have been used for smashing, a convergence with the modern smasher stomatopods. If this is true, they would not hit with the thickened dactylus basis as in extant species, but directly with the anterior edge of the propodus. The sharp-appearing inner edges of the dactylus could then have been used, as in extant smashers, to cut the prey. Convergent evolution of smashers and their presence in Mesozoic times is indicated by certain traces on ammonites [24].

In summary, the species of *Sculda *appear to possess a mixture of morphologies. While the raptorial apparatus is much more similar to that of archaeostomatopods, other characteristics, especially of the pleopods and telson, are already very similar to those of extant stomatopods.

The main discussion of the evolution of the raptorial apparatus is usually based on the second thoracopods. As we have demonstrated, the more posterior ones can also be differentiated and, therefore, cannot be discussed as a series, but must be understood separately. Especially for the species from Lebanon, *Pseudosculda laevis*, *Sculda syriaca *and *Archaeosculda phoenicia*, these appendages still need to be re-investigated. But with the new methods [17] it should be possible to find more information on this issue.

## Conclusions

Morphological details of fossil stomatopods are incompletely known. The species of *Sculda*, especially ?*Sculda pennata/spinosa*, are among the ones known best. The chances of adding further developmental information are regarded as high. Further investigations also on stomatopod specimens from Lebanon have the potential of adding significant information; for example, the specimen illustrated by Schram [8] (his Figure 20) should be re-investigated with the new method of orange-green fluorescence [17].

Compared to the Palaeozoic species, those from the Mesozoic have obviously been understudied in the past. Their preservation is much finer than previously thought and by use of fluorescence technique much easier to distinguish from the matrix compared to older methods. Also small specimens can be found with rather complete preservation of fine details, while for example the fossils from the Mazon Creek fauna are more difficult to investigate as studying them "can be compared sometimes to attempting examination of fine points of anatomy through the bottom of a glass full of water" [8] (p.895). Additionally, the first documentation of developmental data opens a complete new range of possible characters for reconstructing the phylogeny and understanding the evolution of Stomatopoda.

We are optimistic that some of the still open questions concerning the phylogenetic position of the one or other taxon can be solved in the future based on re-investigations of material using new documentary methods. This holds in particular for the phylogenetic status of Pseudosculdidae.

## Methods

### Material

We investigated specimens chosen from a larger collection that will be the basis for a taxonomic revision of the Mesozoic stomatopods. Some specimens are from museum collections (Museum für Naturkunde Stuttgart SMNS, Staatliche Sammlung für Paläontologie und Historische Geologie München SSPHG), but the most impressive specimens are from various private collections (Figure 2, Tab. 1).

### Documentation

The specimens were documented with different methods adapted to the special requirements of each specimen (see table 1). Small specimens were documented using the UV-fluorescence composite imaging method [25] (wavelength ca. 358 nm) or, depending on the autofluorescence capabilities of the specimens, with orange-green fluorescence (wavelength ca. 546 nm) [17]. Larger specimens were documented with composite images under normal light [17,26]. Specimens exhibiting high relief were documented with stereo images to provide spatial information [17]. In some specimens the surrounding matrix was virtually removed using Photoshop CS3. Reconstructed morphologies are presented as 3D virtual models assembled in the freely available 3D modelling software Blender (Figure 8) [17,25-27]. The image of a representative of Unipeltata sensu stricto in Figure 8 is a volume rendering of a CT scan.

### Phylogenetic analysis

The matrix from Schram [8] was taken as a basis for the computer-based phylogenetic analysis. It was altered and amended in a number of ways (see Additional files 2 and 3):

Firstly, all multi-state characters were transformed into a series of binary characters, as favoured by several authors [28,29]. This was done for certain characters that appeared not to be necessarily coupled. Furthermore, this method is seen as having two direct advantages for the current analysis. Multi-state characters do not allow one to identify apomorphic character conditions that are part of a step-wise acquisition. One example is the number of free thoracic segments, i.e., those not hidden under the head shield. Schram [8] coded the various states as a multi-state character (character 1 of [8]):

- all segments hidden under the shield,

- one segment free,

- three segments free, and

- four segments free.

When coding the characters as multi-state, potential apomorphies may be overlooked, as "three segments free" may in fact be a uniting character for those species with three segments free AND four segments free. Unfortunately, these characters may indeed not be completely independent, as for species with the character state "sixth thoracic segment free" the character state "eighth thoracic segment free" is automatically also fulfilled. This may lead to an overestimation of such a character. A similar way of coding can be found in various analyses (e.g., [30] for maxillipeds) Thus, in contrast to the multi-state character coding, which may underestimate certain characters, binary coding may overestimate certain characters. We are aware of this difficulty, but also cannot provide a reliable solution. Hence, our approach was to code binary characters (following [28]) and code as neutrally as possible. As a test tool reciprocal illumination may be used, i.e., the resulting tree can be tested whether the evolutionary scenario arising thereby is plausible or not.

The second advantage is that it becomes possible to code only imperfectly known morphologies more easily. For species where the thorax area is imperfectly known, the multi-state character coding of the free thoracic segments must be coded with a question mark. But with several multi-state characters it is possible to code the information of at least one segment, the eighth thoracic segment (i.e., character 1 as "1" and the others, i.e., characters 2 and 3 with question marks).

Secondly, certain characters used by Schram [8] were excluded from the analysis, because the phrasing of the character states was not apparent to us, therefore, we omitted these characters in our analysis. These characters are:

Character 5: "raptorial limbs inflated". Whether the raptorial limbs are "inflated" or not appears to be difficult to judge in flattened fossils.

Character 12: "armature of 2nd thoracic dactylus". It was unclear to us what the difference between "unarmed" and the two different conditions of "smooth" should be.

Character 13: "pleural spines on pleon". The exact nature of pleural edges is often difficult to judge, especially in flattened fossils. Thus, it is unclear to us when to truly call a structure as being a spine.

Thirdly, the following characters were amended to the analysis:

- Telson length to width larger than 1.5 or stouter (our character 33).

- First raptorial limb differentiated compared to the fourth (our character 34).

- First raptorial limb at least twice as large as fourth (dactylus or propodus length) (our character 35).

- First raptorial limb at least three times as long as fourth (dactylus or propodus length) (our character 36).

The last two characters are again problematic as they are not truly independent, but both morphologies are present within the material and both might be an apomorphic character for a certain group, thus need to be included. Instead of coding extant Unipeltata as showing variable states for certain characters, we coded instead a single extant species, *Squilla mantis*.

Unfortunately, species too imperfectly known, although possibly being of importance, could not be included into the analysis. Although including imperfectly known fossils has been shown to significantly contribute to phylogenetic analyses as long as at least 25% of the characters are known [31], fossils can also destabilise phylogenies when critical characters are missing [32,33]. In the present case the missing information of the tail fan region is critical. The tail fan region is a very important character for stomatopod taxonomy and, as it appears, also for its phylogeny. The imperfectly known morphology prohibited the inclusion of the recently described species *Spinosculda ehrlichi *[15] as well as the single known specimen of a larval stomatopod from the Solnhofen Lithographic Limestones, which also probably represents a new species [25].

The phylogenetic analysis with 20 taxa and 36 binary characters was run in 'PHYLIP pars' (Settings: Search for best tree: Yes; Search option: More thorough search; Number of trees to save: 10,000; Randomize input order of species: No; Use input order; Outgroup root: Yes, at species number 1; Use threshold parsimony: No, use ordinary parsimony; Sites weighted: No; Analyze multiple data sets: No; Input species interleaved: Yes; Terminal type (IBM PC, ANSI, none): ANSI; Print out the data at start of run: No; Print indications of progress of run: Yes; Print out tree: Yes; Print out steps in each site: No; Print character at all nodes of tree: No; Write out trees onto tree file: Yes). As in Schram [8] a euphausiacean was used as outgroup.

## Authors' contributions

JTH carried out the measurements, image processing and the phylogenetic analysis. CH took the image stacks of the specimens and was also involved in image processing. Both JTH and CH drafted the manuscript and contributed equally to this work. VK worked on eumalacostracan uropods in general with special respect to those in stomatopods and helped to draft the manuscript. AM and DW revised the manuscript critically for its scientific content. All authors read and approved the final manuscript.

## Supplementary Material

Additional file 1 **Measured ratios of the raptorial appendages**. Click here for file

Additional file 2 **Characters used for phylogenetic analysis**. (0) = absent, (1) = present, (-) = not applicable, if not stated differently. Click here for file

Additional file 3 **Matrix**. Click here for file

## References

[B1] Kleinlogel S Marshall NJ Electrophysiological evidence for linear polarization sensitivity in the compound eyes of the stomatopod crustacean *Gonodactylus chiragra* The Journal of Experimental Biology 2006 209 4262 4272 10.1242/jeb.02499 17050841

[B2] Chiou T-H Kleinlogel S Cronin T Caldwell R Loeffler B Siddiqi A Goldizen A Marshall J Circular Polarization Vision in a Stomatopod Crustacean Current Biology 2008 18 429 434 10.1016/j.cub.2008.02.066 18356053

[B3] Patek SN Korff WL Caldwell RL Deadly strike mechanism of a mantis shrimp Nature 2004 428 819 820 10.1038/428819a 15103366

[B4] Schram FR An adjustment to the higher taxonomy of the fossil Stomatopoda Crustaceana 2008 81 751 754 10.1163/156854008784513429

[B5] Schram FR Some Middle Pennsylvanian Hoplocarida (Crustacea) and their phylogenetic significance Fieldiana Geology 1969 12 14 235 289

[B6] Schram FR British Carboniferous Malacostraca Fieldiana Geology 1979 40 1 129

[B7] Jenner RA Hof CHJ Schram FR Palaeo- and archaeostomatopods (Hoplocarida, Crustacea) from the Bear Gulch Limestone, Mississipian (Namurian), of Central Montana Contributions to Zoology 1998 67 155 185 http://dpc.uba.uva.nl/ctz/vol67/nr03/art01

[B8] Schram FR Paleozoic proto-mantis shrimp revisited Journal of Paleontology 2007 81 895 916 10.1666/pleo05-075.1

[B9] Hof CHJ Fossil stomatopods (Crustacea: Malacostraca) and their phylogenetic impact Journal of Natural History 1998 32 1567 1576 10.1080/00222939800771101

[B10] Ahyong ST Garassino A Gironi B *Archaeosculda phoenicia *n. gen., n. sp. (Crustacea, Stomatopoda, Pseudosculdidae) from the Upper Cretaceous (Cenomanian) of Lebanon Atti della Società italiana di Scienze naturali e del Museo civico di Storia naturale in Milano 2007 148 I 3 15

[B11] Vega FJ Nyborg T Briceno AR Patarroyo P Luque J Muzquiz HP Stinnesbeck W Upper Cretaceous Crustacea from Mexico and Colombia: Similar faunas and environments during Turonian times Revista Mexicana de Ciencias Geologicas 2007 24 3 403 422

[B12] Kunth A Ueber wenig bekannte Crustaceen von Solenhofen Zeitschrift der Deutschen Geologischen Gesellschaft 1870 22 771 790

[B13] Hof CHJ Briggs DEG Decay and Mineralization of Mantis Shrimps (Stomatopoda: Crustacea) - A Key to Their Fossil Record Palaios 1997 12 420 438 10.2307/3515381

[B14] Schram FR Müller HG Catalog and Bibliography of the Fossil and Recent Stomatopoda 2004 Leiden Backhyus Publishers BV

[B15] Haug C Haug JT Waloszek D Morphology and ontogeny of the Upper Jurassic mantis shrimp *Spinosculda ehrlichi *n. gen. n. sp. from southern Germany Palaeodiversity 2009 2 111 118 http://www.palaeodiversity.org/pdf/02/Pal_2_05_111-118_gu_4c.pdf

[B16] Olesen J Walossek D Limb ontogeny and trunk segmentation in *Nebalia *species (Crustacea, Malacostraca, Leptostraca) Zoomorphology 2000 120 47 64 10.1007/s004350000024

[B17] Haug C Haug JT Waloszek D Maas A Frattigiani R Liebau S New methods to document fossils from lithographic limestones of southern Germany and Lebanon Palaeontologia Electronica 2009 12 3 12 http://palaeo-electronica.org/2009_3/193/index.html 6T

[B18] Feldmann RM Villamil T Kauffman EG Decapod and stomatopod crustaceans from mass mortality lagerstatten: Turonian (Cretaceous) of Colombia Journal of Paleontology 1999 73 91 101

[B19] Förster R Heuschreckenkrebse (Crustacea, Stomatopoda) aus dem Alttertiär von Helmstedt und Handorf (Niedersachsen) und der Oberkreide von Nigeria Neues Jahrbuch für Geologie und Paläontologie Monatshefte 1982 6 321 335

[B20] Morgan SG Goy JW Reproduction and larval development of the mantis shrimp *Gonodactylus bredini *(Crustacea: Stomatopoda) maintained in the laboratory Journal of Crustacean Biology 1987 7 4 595 618 10.2307/1548646

[B21] Schöllmann L Archaeostomatopodea (Malacostraca, Hoplocarida) from the Namurian B (Upper Marsdenian, Carboniferous) of Hagen-Vorhalle (NRW, Germany) and a redescription of some species of the family Tyrannophontidae Geologie und Paläontologie in Westfalen 2004 62 111 141

[B22] Racheboeuf PR Schram FR Vidal M New malacostracan Crustacea from the Carboniferous (Stephanian) lagerstätte of Montceau-Les-Mines, France Journal of Paleontology 2009 83 4 624 629 10.1666/08-171R.1

[B23] Schram FR On Some Pyllocarids and the Origin of the Hoplocarida Fieldiana Geology 1973 26 2 77 94

[B24] Keupp H Sublethal punctures in body chambers of Mesozoic ammonites (forma aegra fenestra n. f.), a tool to interpret synecological relationships, particularly predator-prey interactions Paläontologische Zeitschrift 2006 80 112 123

[B25] Haug JT Haug C Ehrlich M First fossil stomatopod larva (Arthropoda: Crustacea) and a new way of documenting Solnhofen fossils (Upper Jurassic, Southern Germany) Palaeodiversity 2008 1 103 109 http://www.palaeodiversity.org/pdf/01/Palaeodiversity_1_07-103-110.pdf

[B26] Haug JT Haug C Waloszek D Maas A Wulf M Schweigert G Development in Mesozoic scyllarids and implications for the evolution of Achelata (Reptantia, Decapoda, Crustacea) Palaeodiversity 2009 2 97 110 http://www.palaeodiversity.org/pdf/02/Pal_2_04_097-110_gu_4c.pdf

[B27] Haug JT Haug C Maas A Fayers SR Trewin NH Waloszek D Simple 3D images from fossil and Recent micromaterial using light microscopy Journal of Microscopy 2009 233 93 101 10.1111/j.1365-2818.2008.03100.x 19196416

[B28] Pleijel F On character coding for phylogeny reconstruction Cladistics 1995 11 309 315 10.1016/0748-3007(95)90018-7

[B29] Fitzhugh K The philosophical basis of character coding for the inference of phylogenetic hypotheses Zoologica Scripta 2006 35 261 286 10.1111/j.1463-6409.2006.00229.x

[B30] Richter S Scholtz G Phylogenetic analysis of the Malacostraca (Crustacea) Journal of Zoological Systematics and Evolutionary Research 2001 39 113 136 10.1046/j.1439-0469.2001.00164.x

[B31] Santini F Tyler JC The Importance of Even Highly Incomplete Fossil Taxa in Reconstructing the Phylogenetic Relationships of the Tetradontiformes (Acanthomorpha: Pisces) Integrative and Comparative Biology 2004 44 349 357 10.1093/icb/44.5.349 21676720

[B32] Schram FR Hof CHJ Edgecombe GD Fossils and the interrelationships of major crustacean groups Arthropod Fossils and Phylogeny 1998 New York, Columbia University Press 233 302

[B33] Schram FR Dixon CJ Decapod phylogeny: addition of fossil evidence to a robust morphological cladistic data set Bulletin of the Mizunami Fossil Museum 2004 31 1 19

