# Supplementary material for: Evolution of mantis shrimps (Stomatopoda, Malacostraca) in the light of new Mesozoic fossils
Source: BMC Evol Biol. 2010 Sep 21;10:290. doi: 10.1186/1471-2148-10-290 (PMC2955030; doi:10.1186/1471-2148-10-290)
Supplement: Additional file 1 — Measured ratios of the raptorial appendages. [file 1471-2148-10-290-S1.PDF]

Additional File 1 - Measured ratios of the raptorial appendages

A=Length of dactylus of first raptorial limb, set as 1. B=Length of dactylus of second raptorial limb divided by the length of dactylus of first one. C=Length of dactylus of third raptorial limb divided by the length of dactylus of first one. D=Length of dactylus of fourth raptorial limb divided by the length of dactylus of first one. For species marked with asterix (*) measurements are based on images in Schram (2007), therefore given as estimated. Instead of the dactyli, the propodi were measured for these species. Other species were either treated as "differentiated" or "sub-equal".

Species	A	B	C	D
?*Sculda pennata/spinosa*	1	0.87	0.47	0.40
?*Sculda pusilla*	1	0.50	0.29	0.24
Pseudosculda laevis	1	0.29	0.27	0.27
Squilla mantis	1	0.26	0.26	0.26
*Tyrannophontes gigantion**	1	≈0.39	≈0.31	≈0.28
*Tyrannophontes theridion**	1	≈0.54	≈0.30	≈0.21
*Gorgonophontes fraiponti**	1	≈0.57	≈0.5	≈0.5
*Gorgonophontes peleron**	1	≈0.75	≈0.7	≈0.7
