# Supplementary material for: Evolution of mantis shrimps (Stomatopoda, Malacostraca) in the light of new Mesozoic fossils
Source: BMC Evol Biol. 2010 Sep 21;10:290. doi: 10.1186/1471-2148-10-290 (PMC2955030; doi:10.1186/1471-2148-10-290)
Supplement: Additional file 2 — Characters used for phylogenetic analysis. (0) = absent, (1) = present, (-) = not applicable, if not stated differently. [file 1471-2148-10-290-S2.PDF]

Additional file 2 - Characters used for phylogenetic analysis

1. Shield covering thorax segment 8 (in adult).
2. Shield covering thorax segment 7.
3. Shield covering thorax segment 5.
4. Shield rounded in lateral view.
5. Shield laterally enveloping the whole thorax.
6. Subdivided into three fields, one median, two lateral ones.
7. Thorax subdivided into functional units.
8. Raptorial limb portion 5 (“ischium”) equal to portion 3 (carpus) (1) or twice as long (0).
9. Raptorial limb portion 4 (“merus”) long (0) or short (1).
10. Raptorial limb portion 4 (“merus”) with saddle.
11. Saddle of the limb portion 4 (“merus”) incipient (0) or well developed (1).
12. Limb portion 3 (carpus) long (0) or short (1).
13. Jackknife joint present.
14. Jackknife joint between limb portion 4 (“merus”) and 3 (carpus) (0) or portion 3 (carpus) and 2 (propodus) (1).
15. First raptorial limb sub-equal (0) or differentiated (1) compared to second.
16. First raptorial limb at least twice as large as the second.
17. First raptorial limb at least three times the size of the second.
18. First raptorial limb portion 2 (propodus) armed with spines.
19. First raptorial limb portion 2 (propodus) pectinate.
20. Uropod exopod and endopod lobate (0) or blade-like (1).
21. Uropod exopod composed of one element (0) or two (1).
22. Uropod exopod with smooth outer margin (0) or decorated with spines/serrations (1).
23. Uropod endopod simple lobate (0) or reduced with a small tip (1).
24. Uropod with dorsal basipodal process.
25. Uropod with ventral basipodal process.
26. Ventral basipodal process of the uropod enlarged.
27. Telson with median crest or ridge.
28. Telson with submedian crest.
29. Telson with median terminal end.
30. Telson with median process.
31. Median process of the telson sub-triangular (0) or as a long spine (1).

32. Telson with spines along the margin.
33. Telson length to width larger than 1.5 (0) or stouter (1).
34. First raptorial limb differentiated compared to the fourth.
35. First raptorial limb at least twice as large as fourth (dactylus or propodus length).
36. First raptorial limb at least three times as large as fourth (dactylus or propodus length).

Characters used her correspond to characters used by Schram (2007) in the following way (character used in Schram as number, behind them in brackets our corresponding characters): 1(1-3); 2 (4-5); 4(7); 6(8); 7(9-11); 8(12); 9(13-14); 10(15-17); 11(18-19); 14(20-21); 15(22); 16(23); 17(24-26); 18(27); 19(28); 20(29-31); 21(32-33)
