# Supplementary material for: Evolution of mantis shrimps (Stomatopoda, Malacostraca) in the light of new Mesozoic fossils
Source: BMC Evol Biol. 2010 Sep 21;10:290. doi: 10.1186/1471-2148-10-290 (PMC2955030; doi:10.1186/1471-2148-10-290)
Supplement: Additional file 3 — Matrix. [file 1471-2148-10-290-S3.PDF]

Additional file 3 - Matrix

character	1	2	3	4	5	6	7	8	9	10	11	12	13	14	15	16	17	18	19	20	21	22	23	24	25	26	27	28	29	30	31	32	33	34	35	36
outgroup	1	1	1	1	1	0	0	–	–	–	–	0	0	–	–	–	–	–	–	0	1	0	0	0	0	–	0	0	1	0	–	1	0	–	–	–
Kallidecthes richardsoni	1	1	1	1	1	0	0	–	–	–	–	0	0	–	–	–	–	–	–	1	0	0	0	0	0	–	0	0	0	0	–	0	0	–	–	–
Perimecturus rapax	1	1	1	1	1	0	1	?	?	0	–	?	?	?	?	?	?	?	?	1	0	1	1	1	1	0	1	0	1	1	1	1	0	?	?	?
Perimecturus parki	1	1	1	1	1	0	1	0	1	0	–	1	1	1	0	0	0	0	0	1	0	0	1	1	0	–	0	1	1	1	1	1	0	0	0	0
Bairdops elegans	1	1	1	1	1	0	1	0	1	0	–	1	1	0	0	0	0	0	0	1	0	0	1	0	0	–	1	0	1	1	1	1	0	0	0	0
Bairdops beargulchensis	0	1	1	1	1	0	1	0	1	0	–	1	1	0	0	0	0	?	?	1	0	0	?	0	0	–	1	1	1	1	1	1	0	0	0	0
Archaeocaris vermiformis	0	0	1	1	1	0	1	0	1	0	–	0	1	0	0	0	0	0	1	0	1	0	0	0	0	–	0	0	?	?	?	1	0	0	0	0
Archaeocaris graffhami	0	0	1	1	1	0	1	0	1	0	–	0	1	0	0	0	0	?	?	?	?	?	?	0	0	–	0	0	?	?	?	1	0	0	0	0
Gorgonophontes fraiponti	0	0	1	1	0	0	1	0	1	0	–	1	1	0	1	0	0	1	0	0	1	0	0	1	1	0	0	1	1	1	0	1	0	1	0	0
Gorgonophontes peleron	0	0	1	1	0	0	1	0	1	0	–	1	1	0	1	0	0	1	0	0	1	0	0	1	1	0	0	1	1	1	0	1	0	1	0	0
Daidal acanthocercus	0	0	0	1	0	0	1	0	1	0	–	1	1	0	0	0	0	1	0	0	0	1	0	1	1	0	1	1	1	1	0	1	0	0	0	0
Daidal pattoni	0	0	0	1	0	0	1	0	1	0	–	1	1	0	0	0	0	?	?	?	?	?	?	?	?	?	?	1	1	1	0	?	?	0	0	0
Daidal schoellmanni	0	0	0	1	0	0	1	0	1	0	–	1	1	0	0	0	0	1	0	0	0	1	0	1	1	0	1	1	1	1	0	1	0	0	0	0
Tyrannophontes theridion	0	0	0	1	0	0	1	1	0	1	0	1	1	1	1	0	0	1	1	0	0	0	0	1	0	–	0	1	1	1	0	1	0	1	1	1
Tyrannophontes gigantion	0	0	0	1	0	0	1	1	0	1	0	1	1	1	1	1	0	0	1	?	?	?	?	?	?	?	?	?	?	?	?	?	?	1	1	1
Pseudosculda laevis	0	0	0	0	0	1	1	1	0	1	1	1	1	1	1	1	1	1	1	1	0	1	0	0	1	1	1	0	0	0	–	1	1	1	1	1
?*Sculda pennata/spinosa*	0	0	1	0	0	1	1	1	0	?	?	1	1	0	1	0	0	0	0	1	0	1	0	0	1	1	0	0	0	0	–	1	1	1	1	0
?*Sculda pusilla*	0	?	?	0	0	?	1	?	?	?	?	?	?	?	1	1	0	0	?	1	0	1	0	0	1	1	0	0	0	0	–	1	1	1	1	1
Squilla mantis	0	0	0	0	0	1	1	1	0	1	1	1	1	1	1	1	1	1	1	0	1	1	0	0	1	1	0	0	0	0	–	1	1	1	1	1
Chabardella spinosa	0	0	0	1	0	0	1	0	1	?	?	1	1	0	1	0	0	0	0	?	0	1	?	?	?	?	?	?	?	?	?	?	?	1	0	0
